# *catena*-Poly[[tri­phenyl­tin(IV)]-μ-*N*-(4-acetyl­phen­yl)maleamato]

**DOI:** 10.1107/S1600536813017613

**Published:** 2013-07-03

**Authors:** Moazzam H. Bhatti, Uzma Yunus, Nosheen Mussarat, Madeleine Helliwell, Richard Prendergast

**Affiliations:** a Department of Chemistry, Allama Iqbal Open University, Islamabad, Pakistan; b School of Chemistry, University of Manchester, Manchester M30 9PL, England

## Abstract

The crystal structure of the polymeric title compound, [Sn(C_6_H_5_)_3_(C_12_H_10_NO_4_)]_*n*_, comprises polymeric chains whereby adjacent Sn atoms are bridged by carboxyl­ate and amide carbonyl O atoms [Sn—O = 2.115 (15) and 2.653 (1) Å, respectively]. The Sn^IV^ atom is five-coordinated showing a distorted trigonal–bipyramid geometry, with the three phenyl *ipso*-C atoms defining the trigonal plane and the axial positions occupied by O atoms [O—Sn—O = 171.91 (5)°]. Intra­molecular N—H⋯O hydrogen bonding leads to a seven-membered loop. There is an intra­molecular C—H⋯O inter­action within the polymeric chain. An inter­molecular C—H⋯O inter­action along *c* links the polymeric chains into sheets which are linked into a three-dimensional network *via* C—H⋯π inter­actions.

## Related literature

For reviews of organotin structural chemistry, see: Tiekink (1991 ▶, 1994 ▶). For related structures, see: Sadiq-ur-Rehman *et al.* (2005 ▶); Parvez *et al.* (2002 ▶).
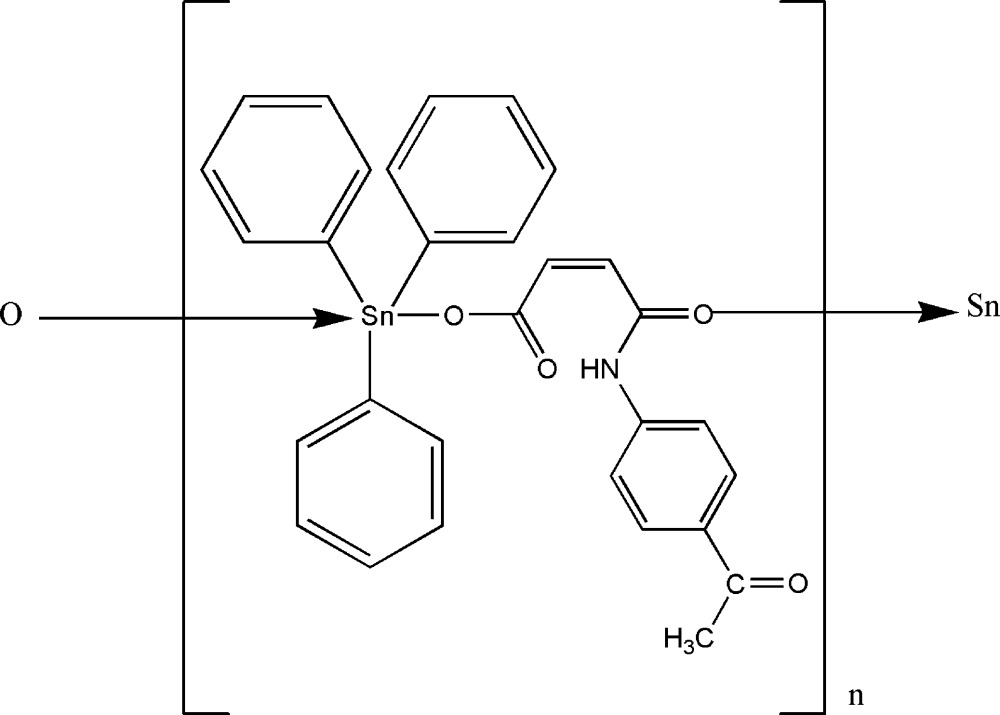


## Experimental

### 

#### Crystal data

[Sn(C_6_H_5_)_3_(C_12_H_10_NO_4_)]
*M* *_r_* = 582.20
Triclinic, 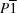

*a* = 9.7556 (5) Å
*b* = 11.3298 (6) Å
*c* = 12.0571 (6) Å
α = 73.187 (1)°
β = 87.082 (1)°
γ = 79.841 (1)°
*V* = 1255.69 (11) Å^3^
*Z* = 2
Mo *K*α radiation
μ = 1.05 mm^−1^
*T* = 100 K
0.40 × 0.30 × 0.30 mm

#### Data collection

Bruker SMART CCD area-detector diffractometer
Absorption correction: multi-scan (*SADABS*; Bruker, 2001 ▶) *T*_min_ = 0.726, *T*_max_ = 1.000
10025 measured reflections
5034 independent reflections
4905 reflections with *I* > 2σ(*I*)
*R*_int_ = 0.017

#### Refinement

*R*[*F* ^2^ > 2σ(*F* ^2^)] = 0.024
*wR*(*F* ^2^) = 0.061
*S* = 1.09
5034 reflections
425 parameters
All H-atom parameters refined
Δρ_max_ = 0.94 e Å^−3^
Δρ_min_ = −0.52 e Å^−3^

### 

Data collection: *SMART* (Bruker, 2001 ▶); cell refinement: *SAINT* (Bruker, 2002 ▶); data reduction: *SAINT*; program(s) used to solve structure: *SHELXS97* (Sheldrick, 2008 ▶); program(s) used to refine structure: *SHELXL97* (Sheldrick, 2008 ▶); molecular graphics: *SHELXTL* (Sheldrick, 2008 ▶) and *PLATON* (Spek, 2009 ▶); software used to prepare material for publication: *SHELXTL*.

## Supplementary Material

Crystal structure: contains datablock(s) global, I. DOI: 10.1107/S1600536813017613/tk5234sup1.cif

Structure factors: contains datablock(s) I. DOI: 10.1107/S1600536813017613/tk5234Isup2.hkl

Additional supplementary materials:  crystallographic information; 3D view; checkCIF report

## Figures and Tables

**Table 1:** Hydrogen-bond geometry (Å, °) *Cg*1–*Cg*3 are the centroids of the C1–C6, C7–C12 and C13–C18 benzene rings, respectively.

*D*—H⋯*A*	*D*—H	H⋯*A*	*D*⋯*A*	*D*—H⋯*A*
N1—H1*N*⋯O2	0.87 (3)	1.88 (3)	2.707 (2)	158 (2)
C6—H6⋯O3^i^	0.91 (3)	2.58 (3)	3.253 (3)	131 (2)
C20—H20⋯O4^ii^	0.93 (3)	2.48 (3)	3.242 (3)	139 (2)
C3—H3⋯*Cg*2^iii^	0.92 (3)	2.93 (3)	3.651 (2)	137 (2)
C27—H27⋯*Cg*3^iv^	0.92 (3)	2.83 (3)	3.674 (2)	154 (2)
C30—H30*C*⋯*Cg*1^v^	0.93 (3)	2.93 (3)	3.794 (4)	155 (3)

Symmetry codes: (i) 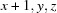; (ii) 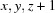; (iii) 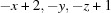; (iv) 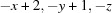; (v) 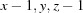.

**Fig. 1.**
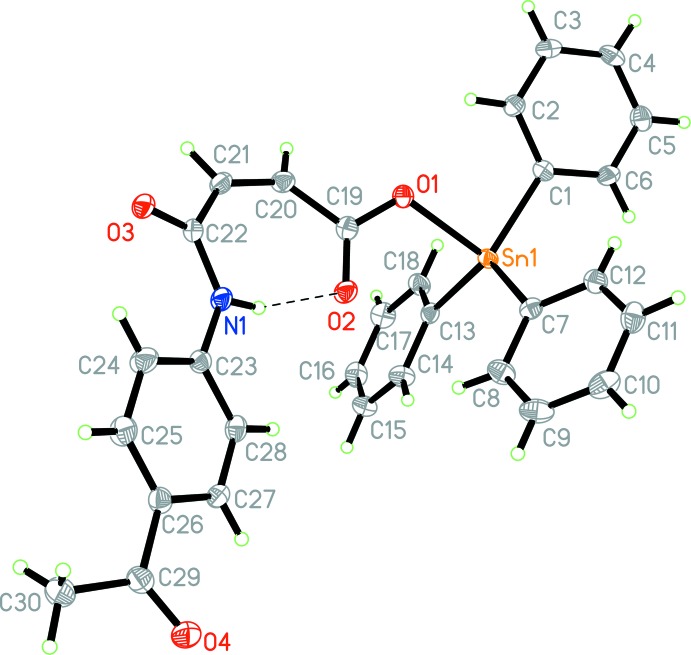
The asymmetric unit of (I) with atomic numbering scheme. Displacement ellipsoids are drawn at the 50% probability level.

**Fig. 2.**
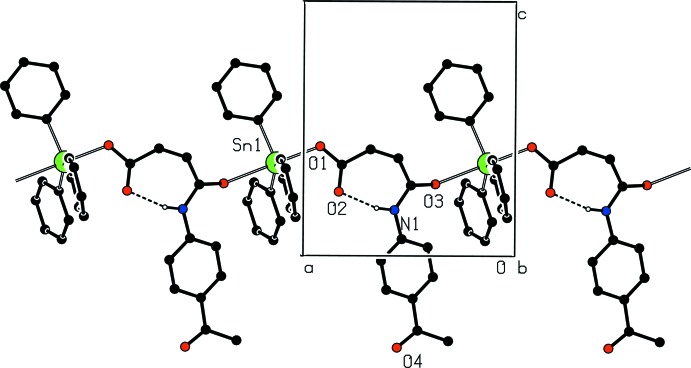
Polymeric chains viewed down b. The intramolecular N1—H1N···O2 hydrogen bonds are shown with dashed lines.

## supplementary crystallographic information

### Comment

Organotin(IV) carboxylates are well known for their potential applications and rich structural diversity (Tiekink, 1991). Among organotin(IV) carboxylates, tri-organotin(IV) carboxylates have shown discrete to polymer chain structures (Tiekink, 1994). Triphenyltin(IV) carboxylates generally adopt a tetrahedral geometry with a monodentate carboxylate [Sadiq-ur-Rehman *et al.*, 2005] or a distorted trigonal bipyramidal geometry with bridging ligand or with an additional donor atom like N or O derived from the adjacent ligand [Parvez *et al.*, 2002]. In the present report we have studied the polymeric title compound, (I), *catena*-poly{[triphenyltin(IV)]-*µ*-*N*-(4-acetylphenyl)maleamato}, (Fig. 1).

The geometry around tin atom is distorted trigonal bipyramidal as defined by three *ipso*-carbon atoms of the three phenyl rings in equatorial positions [Sn1—C1 = 2.133 (2) Å, Sn1—C7 = 2.117 (2) Å, Sn1—C13 = 2.119 (2) Å] while the axial positions are occupied by the O atom of a monodentate carboxylate ligand [Sn1—O1 = 2.115 (15) Å] and the amide carbonyl of an adjacent ligand [Sn1—O3 = 2.653 (1) Å]. The sum of the angles in the equatorial plane is 354.9° indicating distortion. The major distortion from ideal trigonal bipyramidal geometry is found in the axial angle [O1—Sn1—O3 = 171.91 (5)°]. The monodentate mode of coordination of the carboxylate ligand is reflected in the disparate O1—C19 and O2—C19 bond distances of 1.295 (3) and 1.234 (3) Å, respectively, with the longer separation associated with the stronger Sn1—O1 interaction. The much longer distance, Sn1—O2 = 3.100 (1) Å, further demonstrates the monodentate nature of the carboxylate ligand. Although this value is quite long for any coordination of Sn with O, it falls well within the sum of their van der Waals radii (3.70 Å). Adjacent Sn atoms are linked by the O1 and O3 atoms of each ligand generating polymeric chains (Fig. 2).

The ligand forms an essentially planar seven-membered ring involving an intramolecular N1—H1N···O2 hydrogen bond (Figs 1 and 2).

### Experimental

A solution of maleic anhydride (1.0 g, 10 mmol) in ethyl acetate (50 ml ) was added to a solution of *p*-aminoacetophonene (1.35 g, 10 mmol) in ethyl acetate (50 ml) in a 250 ml conical flask. The mixture was stirred for 3 h at room temperature. After stirring, the precipitates of the acid were filtered and recrystallized with ethanol. Equimolar amounts of acid (2.33 g, 10 mmol) and triphenyltin hydroxide (3.17 g 10.0 mmol) were suspended in a dry ethanol / acetone (100 ml, 8:2) solvent mixture and refluxed for 8 h. After cooling to room temperature, the reaction mixture was filtered and solvents were evaporated in rotary evaporator. The solid obtained was recrystallized from chloroform with few drops of *n*-hexane.

### Refinement

H atoms were found by difference Fourier techniques and refined isotropically.

### Crystal data

**Table:**

[Sn(C_6_H_5_)_3_(C_12_H_10_NO_4_)]	*Z* = 2
*M**_r_* = 582.20	*F*(000) = 588
Triclinic, *P*1	*D*_x_ = 1.540 Mg m^−^^3^
Hall symbol: -P 1	Mo *K*α radiation, λ = 0.71073 Å
*a* = 9.7556 (5) Å	Cell parameters from 7807 reflections
*b* = 11.3298 (6) Å	θ = 2.2–26.3°
*c* = 12.0571 (6) Å	µ = 1.05 mm^−^^1^
α = 73.187 (1)°	*T* = 100 K
β = 87.082 (1)°	Block, colourless
γ = 79.841 (1)°	0.40 × 0.30 × 0.30 mm
*V* = 1255.69 (11) Å^3^	

### Data collection

**Table:**

Bruker SMART CCD area-detector diffractometer	5034 independent reflections
Radiation source: fine-focus sealed tube	4905 reflections with *I* > 2σ(*I*)
Graphite monochromator	*R*_int_ = 0.017
φ and ω scans	θ_max_ = 26.4°, θ_min_ = 1.9°
Absorption correction: multi-scan (*SADABS*; Bruker, 2001)	*h* = −12→12
*T*_min_ = 0.726, *T*_max_ = 1.000	*k* = −14→14
10025 measured reflections	*l* = −14→14

### Refinement

**Table:**

Refinement on *F*^2^	Primary atom site location: structure-invariant direct methods
Least-squares matrix: full	Secondary atom site location: difference Fourier map
*R*[*F*^2^ > 2σ(*F*^2^)] = 0.024	Hydrogen site location: inferred from neighbouring sites
*wR*(*F*^2^) = 0.061	All H-atom parameters refined
*S* = 1.09	*w* = 1/[σ^2^(*F*_o_^2^) + (0.0344*P*)^2^ + 0.7706*P*] where *P* = (*F*_o_^2^ + 2*F*_c_^2^)/3
5034 reflections	(Δ/σ)_max_ = 0.002
425 parameters	Δρ_max_ = 0.94 e Å^−^^3^
0 restraints	Δρ_min_ = −0.52 e Å^−^^3^

### Special details

**Table:**

Geometry. All e.s.d.'s (except the e.s.d. in the dihedral angle between two l.s. planes) are estimated using the full covariance matrix. The cell e.s.d.'s are taken into account individually in the estimation of e.s.d.'s in distances, angles and torsion angles; correlations between e.s.d.'s in cell parameters are only used when they are defined by crystal symmetry. An approximate (isotropic) treatment of cell e.s.d.'s is used for estimating e.s.d.'s involving l.s. planes.
Refinement. Refinement of *F*^2^ against ALL reflections. The weighted *R*-factor *wR* and goodness of fit *S* are based on *F*^2^, conventional *R*-factors *R* are based on *F*, with *F* set to zero for negative *F*^2^. The threshold expression of *F*^2^ > σ(*F*^2^) is used only for calculating *R*-factors(gt) *etc*. and is not relevant to the choice of reflections for refinement. *R*-factors based on *F*^2^ are statistically about twice as large as those based on *F*, and *R*- factors based on ALL data will be even larger.

### Fractional atomic coordinates and isotropic or equivalent isotropic displacement parameters (Å^2^)

**Table:**

	*x*	*y*	*z*	*U*_iso_*/*U*_eq_	
Sn1	1.118867 (13)	0.216792 (11)	0.364576 (10)	0.01473 (6)	
O1	0.91398 (14)	0.21171 (14)	0.43008 (12)	0.0192 (3)	
O2	0.82580 (15)	0.29679 (14)	0.25203 (12)	0.0203 (3)	
O3	0.36940 (15)	0.25668 (14)	0.28265 (12)	0.0207 (3)	
O4	0.54744 (17)	0.37815 (17)	−0.35968 (13)	0.0294 (4)	
N1	0.56244 (18)	0.31413 (16)	0.18064 (15)	0.0180 (3)	
C1	1.2068 (2)	0.12405 (18)	0.53210 (17)	0.0157 (4)	
C2	1.1190 (2)	0.09341 (19)	0.62727 (18)	0.0188 (4)	
C3	1.1730 (2)	0.0327 (2)	0.73784 (18)	0.0223 (4)	
C4	1.3154 (2)	0.0025 (2)	0.75508 (18)	0.0229 (4)	
C5	1.4035 (2)	0.0305 (2)	0.6616 (2)	0.0249 (5)	
C6	1.3499 (2)	0.0898 (2)	0.55069 (19)	0.0214 (4)	
C7	1.1471 (2)	0.10390 (19)	0.24991 (17)	0.0163 (4)	
C8	1.0993 (2)	0.1477 (2)	0.13559 (19)	0.0228 (4)	
C9	1.1310 (3)	0.0744 (2)	0.0605 (2)	0.0261 (5)	
C10	1.2088 (2)	−0.0445 (2)	0.0984 (2)	0.0256 (5)	
C11	1.2536 (2)	−0.0907 (2)	0.2129 (2)	0.0246 (5)	
C12	1.2232 (2)	−0.0165 (2)	0.28751 (19)	0.0199 (4)	
C13	1.0903 (2)	0.41459 (18)	0.30166 (17)	0.0163 (4)	
C14	1.0550 (2)	0.4792 (2)	0.18678 (19)	0.0227 (4)	
C15	1.0324 (2)	0.6089 (2)	0.15076 (19)	0.0246 (5)	
C16	1.0452 (2)	0.6755 (2)	0.2282 (2)	0.0239 (4)	
C17	1.0806 (2)	0.6130 (2)	0.3421 (2)	0.0248 (5)	
C18	1.1028 (2)	0.4831 (2)	0.37833 (19)	0.0210 (4)	
C19	0.8119 (2)	0.26463 (19)	0.35832 (18)	0.0176 (4)	
C20	0.6763 (2)	0.2894 (2)	0.41850 (18)	0.0195 (4)	
C21	0.5453 (2)	0.3011 (2)	0.38405 (18)	0.0190 (4)	
C22	0.4852 (2)	0.28728 (18)	0.27760 (17)	0.0172 (4)	
C23	0.5275 (2)	0.32237 (19)	0.06658 (17)	0.0172 (4)	
C24	0.4041 (2)	0.2941 (2)	0.0351 (2)	0.0259 (5)	
C25	0.3785 (2)	0.3122 (2)	−0.0815 (2)	0.0258 (5)	
C26	0.4739 (2)	0.35404 (19)	−0.16698 (18)	0.0193 (4)	
C27	0.5990 (2)	0.3783 (2)	−0.13311 (18)	0.0195 (4)	
C28	0.6248 (2)	0.36391 (19)	−0.01899 (19)	0.0194 (4)	
C29	0.4505 (2)	0.3716 (2)	−0.29234 (18)	0.0218 (4)	
C30	0.3051 (3)	0.3806 (3)	−0.3335 (2)	0.0289 (5)	
H2	1.023 (3)	0.112 (2)	0.618 (2)	0.023 (6)*	
H3	1.112 (3)	0.013 (3)	0.798 (2)	0.032 (7)*	
H4	1.354 (3)	−0.036 (3)	0.826 (3)	0.033 (7)*	
H5	1.498 (3)	0.004 (3)	0.672 (2)	0.032 (7)*	
H6	1.409 (3)	0.107 (2)	0.489 (2)	0.027 (7)*	
H8	1.047 (3)	0.227 (3)	0.113 (2)	0.024 (6)*	
H9	1.104 (3)	0.105 (3)	−0.011 (3)	0.042 (8)*	
H10	1.228 (3)	−0.092 (3)	0.049 (2)	0.031 (7)*	
H11	1.304 (3)	−0.170 (3)	0.240 (2)	0.026 (7)*	
H12	1.254 (3)	−0.046 (2)	0.357 (2)	0.020 (6)*	
H14	1.045 (3)	0.436 (3)	0.132 (2)	0.030 (7)*	
H15	1.010 (3)	0.650 (3)	0.075 (2)	0.030 (7)*	
H16	1.032 (3)	0.756 (2)	0.206 (2)	0.020 (6)*	
H17	1.085 (3)	0.654 (3)	0.393 (2)	0.030 (7)*	
H18	1.127 (3)	0.442 (2)	0.453 (2)	0.026 (6)*	
H20	0.688 (3)	0.302 (2)	0.489 (2)	0.021 (6)*	
H21	0.476 (3)	0.319 (2)	0.437 (2)	0.022 (6)*	
H24	0.339 (3)	0.263 (3)	0.091 (3)	0.036 (7)*	
H25	0.301 (3)	0.293 (3)	−0.103 (2)	0.037 (8)*	
H27	0.663 (3)	0.408 (3)	−0.188 (2)	0.032 (7)*	
H28	0.711 (3)	0.385 (3)	0.001 (2)	0.031 (7)*	
H30A	0.303 (3)	0.410 (3)	−0.412 (3)	0.045 (8)*	
H30B	0.244 (3)	0.434 (3)	−0.300 (2)	0.027 (7)*	
H30C	0.282 (3)	0.301 (3)	−0.315 (3)	0.037 (8)*	
H1N	0.647 (3)	0.326 (2)	0.188 (2)	0.025 (7)*	

### Atomic displacement parameters (Å^2^)

**Table:**

	*U*^11^	*U*^22^	*U*^33^	*U*^12^	*U*^13^	*U*^23^
Sn1	0.01521 (8)	0.01594 (8)	0.01313 (8)	−0.00281 (5)	−0.00159 (5)	−0.00392 (5)
O1	0.0137 (7)	0.0241 (7)	0.0190 (7)	−0.0039 (6)	−0.0019 (5)	−0.0040 (6)
O2	0.0153 (7)	0.0291 (8)	0.0178 (7)	−0.0057 (6)	0.0007 (5)	−0.0075 (6)
O3	0.0140 (7)	0.0280 (8)	0.0195 (7)	−0.0051 (6)	0.0003 (5)	−0.0052 (6)
O4	0.0261 (8)	0.0426 (10)	0.0191 (8)	−0.0003 (7)	0.0007 (6)	−0.0114 (7)
N1	0.0129 (8)	0.0242 (9)	0.0178 (8)	−0.0044 (7)	−0.0005 (6)	−0.0067 (7)
C1	0.0183 (10)	0.0133 (9)	0.0165 (9)	−0.0044 (7)	−0.0015 (7)	−0.0044 (7)
C2	0.0162 (10)	0.0197 (10)	0.0203 (10)	−0.0049 (8)	0.0007 (8)	−0.0045 (8)
C3	0.0267 (11)	0.0232 (11)	0.0164 (10)	−0.0081 (9)	0.0048 (8)	−0.0032 (8)
C4	0.0299 (12)	0.0211 (10)	0.0151 (10)	−0.0025 (9)	−0.0057 (8)	−0.0010 (8)
C5	0.0172 (11)	0.0305 (12)	0.0247 (11)	−0.0021 (9)	−0.0055 (8)	−0.0046 (9)
C6	0.0182 (10)	0.0252 (11)	0.0191 (10)	−0.0057 (8)	0.0032 (8)	−0.0031 (8)
C7	0.0147 (9)	0.0191 (10)	0.0173 (9)	−0.0069 (7)	0.0013 (7)	−0.0064 (8)
C8	0.0291 (11)	0.0179 (10)	0.0210 (10)	−0.0053 (9)	−0.0030 (9)	−0.0037 (8)
C9	0.0374 (13)	0.0270 (11)	0.0174 (11)	−0.0131 (10)	−0.0009 (9)	−0.0072 (9)
C10	0.0305 (12)	0.0261 (11)	0.0266 (11)	−0.0110 (9)	0.0071 (9)	−0.0150 (10)
C11	0.0212 (11)	0.0204 (11)	0.0337 (12)	−0.0028 (9)	0.0012 (9)	−0.0106 (9)
C12	0.0163 (10)	0.0227 (11)	0.0204 (11)	−0.0050 (8)	−0.0016 (8)	−0.0043 (8)
C13	0.0124 (9)	0.0170 (9)	0.0192 (10)	−0.0032 (7)	0.0009 (7)	−0.0044 (8)
C14	0.0280 (11)	0.0222 (11)	0.0183 (10)	−0.0059 (9)	−0.0001 (8)	−0.0055 (8)
C15	0.0273 (11)	0.0231 (11)	0.0194 (11)	−0.0036 (9)	0.0000 (9)	−0.0001 (9)
C16	0.0217 (11)	0.0169 (11)	0.0320 (12)	−0.0046 (8)	0.0009 (9)	−0.0047 (9)
C17	0.0263 (11)	0.0247 (11)	0.0282 (12)	−0.0074 (9)	−0.0019 (9)	−0.0127 (9)
C18	0.0204 (10)	0.0238 (11)	0.0188 (10)	−0.0034 (8)	−0.0042 (8)	−0.0056 (8)
C19	0.0160 (9)	0.0189 (10)	0.0200 (10)	−0.0051 (8)	−0.0015 (8)	−0.0074 (8)
C20	0.0202 (10)	0.0229 (10)	0.0163 (10)	−0.0048 (8)	0.0004 (8)	−0.0062 (8)
C21	0.0172 (10)	0.0228 (10)	0.0175 (10)	−0.0034 (8)	0.0034 (8)	−0.0069 (8)
C22	0.0144 (9)	0.0174 (9)	0.0183 (10)	0.0000 (7)	−0.0001 (7)	−0.0044 (8)
C23	0.0157 (9)	0.0196 (10)	0.0167 (9)	−0.0023 (8)	−0.0008 (7)	−0.0061 (8)
C24	0.0189 (10)	0.0395 (13)	0.0211 (11)	−0.0116 (9)	0.0017 (8)	−0.0080 (10)
C25	0.0173 (10)	0.0388 (13)	0.0250 (11)	−0.0103 (9)	−0.0032 (8)	−0.0107 (10)
C26	0.0183 (10)	0.0199 (10)	0.0199 (10)	−0.0009 (8)	−0.0015 (8)	−0.0073 (8)
C27	0.0160 (10)	0.0237 (10)	0.0186 (10)	−0.0026 (8)	0.0024 (8)	−0.0065 (8)
C28	0.0136 (9)	0.0215 (10)	0.0240 (11)	−0.0046 (8)	−0.0007 (8)	−0.0069 (8)
C29	0.0247 (11)	0.0210 (10)	0.0200 (10)	−0.0001 (8)	−0.0023 (8)	−0.0079 (8)
C30	0.0264 (12)	0.0409 (14)	0.0217 (12)	−0.0038 (10)	−0.0046 (9)	−0.0130 (11)

### Geometric parameters (Å, º)

**Table:**

Sn1—O1	2.1156 (14)	C12—H12	0.85 (3)
Sn1—C7	2.117 (2)	C13—C18	1.390 (3)
Sn1—C13	2.120 (2)	C13—C14	1.395 (3)
Sn1—C1	2.1331 (19)	C14—C15	1.387 (3)
O1—C19	1.295 (2)	C14—H14	0.95 (3)
O2—C19	1.234 (3)	C15—C16	1.382 (3)
O3—C22	1.233 (2)	C15—H15	0.92 (3)
O4—C29	1.213 (3)	C16—C17	1.380 (3)
N1—C22	1.346 (3)	C16—H16	0.86 (3)
N1—C23	1.406 (3)	C17—C18	1.388 (3)
N1—H1N	0.87 (3)	C17—H17	0.88 (3)
C1—C6	1.394 (3)	C18—H18	0.91 (3)
C1—C2	1.395 (3)	C19—C20	1.498 (3)
C2—C3	1.393 (3)	C20—C21	1.336 (3)
C2—H2	0.93 (3)	C20—H20	0.93 (3)
C3—C4	1.382 (3)	C21—C22	1.497 (3)
C3—H3	0.92 (3)	C21—H21	0.94 (3)
C4—C5	1.379 (3)	C23—C24	1.396 (3)
C4—H4	0.90 (3)	C23—C28	1.401 (3)
C5—C6	1.393 (3)	C24—C25	1.390 (3)
C5—H5	0.92 (3)	C24—H24	0.94 (3)
C6—H6	0.91 (3)	C25—C26	1.388 (3)
C7—C12	1.392 (3)	C25—H25	0.89 (3)
C7—C8	1.397 (3)	C26—C27	1.402 (3)
C8—C9	1.386 (3)	C26—C29	1.490 (3)
C8—H8	0.92 (3)	C27—C28	1.369 (3)
C9—C10	1.385 (3)	C27—H27	0.92 (3)
C9—H9	0.87 (3)	C28—H28	0.97 (3)
C10—C11	1.390 (3)	C29—C30	1.504 (3)
C10—H10	0.90 (3)	C30—H30A	0.90 (3)
C11—C12	1.388 (3)	C30—H30B	0.93 (3)
C11—H11	0.91 (3)	C30—H30C	0.93 (3)
			
O1—Sn1—C7	106.04 (6)	C16—C15—C14	120.3 (2)
O1—Sn1—C13	94.72 (6)	C16—C15—H15	120.2 (17)
C7—Sn1—C13	121.26 (8)	C14—C15—H15	119.4 (17)
O1—Sn1—C1	91.79 (7)	C17—C16—C15	120.0 (2)
C7—Sn1—C1	113.71 (7)	C17—C16—H16	119.3 (16)
C13—Sn1—C1	119.95 (7)	C15—C16—H16	120.7 (16)
C19—O1—Sn1	117.65 (13)	C16—C17—C18	119.7 (2)
C22—N1—C23	128.88 (18)	C16—C17—H17	121.0 (18)
C22—N1—H1N	117.3 (17)	C18—C17—H17	119.2 (18)
C23—N1—H1N	113.8 (17)	C17—C18—C13	121.1 (2)
C6—C1—C2	117.95 (19)	C17—C18—H18	119.7 (17)
C6—C1—Sn1	122.54 (15)	C13—C18—H18	119.3 (17)
C2—C1—Sn1	119.49 (15)	O2—C19—O1	123.74 (19)
C3—C2—C1	120.93 (19)	O2—C19—C20	123.54 (18)
C3—C2—H2	118.3 (16)	O1—C19—C20	112.62 (17)
C1—C2—H2	120.8 (16)	C21—C20—C19	130.75 (19)
C4—C3—C2	120.3 (2)	C21—C20—H20	116.9 (15)
C4—C3—H3	121.6 (17)	C19—C20—H20	112.3 (15)
C2—C3—H3	118.2 (17)	C20—C21—C22	132.41 (19)
C5—C4—C3	119.4 (2)	C20—C21—H21	115.4 (15)
C5—C4—H4	118.2 (18)	C22—C21—H21	112.2 (15)
C3—C4—H4	122.3 (18)	O3—C22—N1	124.60 (19)
C4—C5—C6	120.5 (2)	O3—C22—C21	119.31 (18)
C4—C5—H5	119.5 (17)	N1—C22—C21	116.05 (18)
C6—C5—H5	119.9 (17)	C24—C23—C28	119.58 (19)
C5—C6—C1	120.9 (2)	C24—C23—N1	124.44 (19)
C5—C6—H6	119.6 (17)	C28—C23—N1	115.97 (18)
C1—C6—H6	119.5 (17)	C25—C24—C23	118.9 (2)
C12—C7—C8	118.41 (19)	C25—C24—H24	119.9 (18)
C12—C7—Sn1	118.82 (15)	C23—C24—H24	121.2 (18)
C8—C7—Sn1	122.66 (15)	C26—C25—C24	121.9 (2)
C9—C8—C7	120.7 (2)	C26—C25—H25	118.3 (19)
C9—C8—H8	122.1 (16)	C24—C25—H25	119.8 (19)
C7—C8—H8	117.2 (16)	C25—C26—C27	118.27 (19)
C10—C9—C8	120.4 (2)	C25—C26—C29	122.99 (19)
C10—C9—H9	120 (2)	C27—C26—C29	118.72 (19)
C8—C9—H9	119 (2)	C28—C27—C26	120.75 (19)
C9—C10—C11	119.6 (2)	C28—C27—H27	118.6 (17)
C9—C10—H10	119.5 (18)	C26—C27—H27	120.6 (17)
C11—C10—H10	120.9 (18)	C27—C28—C23	120.56 (19)
C12—C11—C10	119.9 (2)	C27—C28—H28	118.7 (16)
C12—C11—H11	119.1 (16)	C23—C28—H28	120.7 (16)
C10—C11—H11	121.0 (16)	O4—C29—C26	120.4 (2)
C11—C12—C7	121.0 (2)	O4—C29—C30	120.8 (2)
C11—C12—H12	118.1 (17)	C26—C29—C30	118.85 (19)
C7—C12—H12	120.9 (17)	C29—C30—H30A	109 (2)
C18—C13—C14	118.53 (19)	C29—C30—H30B	108.8 (16)
C18—C13—Sn1	118.94 (15)	H30A—C30—H30B	111 (3)
C14—C13—Sn1	122.50 (15)	C29—C30—H30C	109.3 (18)
C15—C14—C13	120.3 (2)	H30A—C30—H30C	107 (3)
C15—C14—H14	118.4 (16)	H30B—C30—H30C	112 (2)
C13—C14—H14	121.2 (16)		
			
C7—Sn1—O1—C19	−67.36 (15)	C7—Sn1—C13—C14	16.1 (2)
C13—Sn1—O1—C19	57.08 (15)	C1—Sn1—C13—C14	169.33 (16)
C1—Sn1—O1—C19	177.32 (14)	C18—C13—C14—C15	−0.3 (3)
O1—Sn1—C1—C6	176.43 (17)	Sn1—C13—C14—C15	177.59 (16)
C7—Sn1—C1—C6	68.03 (18)	C13—C14—C15—C16	0.3 (3)
C13—Sn1—C1—C6	−87.08 (18)	C14—C15—C16—C17	0.0 (3)
O1—Sn1—C1—C2	−2.16 (16)	C15—C16—C17—C18	−0.2 (3)
C7—Sn1—C1—C2	−110.56 (16)	C16—C17—C18—C13	0.2 (3)
C13—Sn1—C1—C2	94.33 (16)	C14—C13—C18—C17	0.1 (3)
C6—C1—C2—C3	1.3 (3)	Sn1—C13—C18—C17	−177.87 (16)
Sn1—C1—C2—C3	179.92 (16)	Sn1—O1—C19—O2	13.9 (3)
C1—C2—C3—C4	0.5 (3)	Sn1—O1—C19—C20	−162.73 (13)
C2—C3—C4—C5	−1.4 (3)	O2—C19—C20—C21	29.0 (4)
C3—C4—C5—C6	0.6 (3)	O1—C19—C20—C21	−154.3 (2)
C4—C5—C6—C1	1.1 (3)	C19—C20—C21—C22	3.8 (4)
C2—C1—C6—C5	−2.1 (3)	C23—N1—C22—O3	5.0 (3)
Sn1—C1—C6—C5	179.34 (17)	C23—N1—C22—C21	−172.72 (19)
O1—Sn1—C7—C12	−102.06 (16)	C20—C21—C22—O3	153.0 (2)
C13—Sn1—C7—C12	152.00 (15)	C20—C21—C22—N1	−29.2 (3)
C1—Sn1—C7—C12	−2.74 (18)	C22—N1—C23—C24	−4.9 (3)
O1—Sn1—C7—C8	81.76 (17)	C22—N1—C23—C28	174.1 (2)
C13—Sn1—C7—C8	−24.2 (2)	C28—C23—C24—C25	−2.2 (3)
C1—Sn1—C7—C8	−178.92 (16)	N1—C23—C24—C25	176.7 (2)
C12—C7—C8—C9	−2.1 (3)	C23—C24—C25—C26	1.9 (4)
Sn1—C7—C8—C9	174.13 (17)	C24—C25—C26—C27	0.0 (3)
C7—C8—C9—C10	1.0 (3)	C24—C25—C26—C29	178.7 (2)
C8—C9—C10—C11	0.8 (3)	C25—C26—C27—C28	−1.5 (3)
C9—C10—C11—C12	−1.7 (3)	C29—C26—C27—C28	179.74 (19)
C10—C11—C12—C7	0.6 (3)	C26—C27—C28—C23	1.2 (3)
C8—C7—C12—C11	1.2 (3)	C24—C23—C28—C27	0.7 (3)
Sn1—C7—C12—C11	−175.11 (16)	N1—C23—C28—C27	−178.29 (19)
O1—Sn1—C13—C18	82.01 (16)	C25—C26—C29—O4	−161.8 (2)
C7—Sn1—C13—C18	−166.00 (15)	C27—C26—C29—O4	16.8 (3)
C1—Sn1—C13—C18	−12.79 (19)	C25—C26—C29—C30	17.9 (3)
O1—Sn1—C13—C14	−95.87 (17)	C27—C26—C29—C30	−163.4 (2)

### Hydrogen-bond geometry (Å, º)

Cg1–Cg3 are the centroids of the C1–C6, C7–C12 and C13–C18 benzene rings, respectively.

**Table:**

*D*—H···*A*	*D*—H	H···*A*	*D*···*A*	*D*—H···*A*
N1—H1*N*···O2	0.87 (3)	1.88 (3)	2.707 (2)	158 (2)
C6—H6···O3^i^	0.91 (3)	2.58 (3)	3.253 (3)	131 (2)
C20—H20···O4^ii^	0.93 (3)	2.48 (3)	3.242 (3)	139 (2)
C3—H3···*Cg*2^iii^	0.92 (3)	2.93 (3)	3.651 (2)	137 (2)
C27—H27···*Cg*3^iv^	0.92 (3)	2.83 (3)	3.674 (2)	154 (2)
C30—H30*C*···*Cg*1^v^	0.93 (3)	2.93 (3)	3.794 (4)	155 (3)

Symmetry codes: (i) *x*+1, *y*, *z*; (ii) *x*, *y*, *z*+1; (iii) −*x*+2, −*y*, −*z*+1; (iv) −*x*+2, −*y*+1, −*z*; (v) *x*−1, *y*, *z*−1.
